# Development of Viscoelastic Multi-Body Simulation and Impact Response Analysis of a Ballasted Railway Track under Cyclic Loading

**DOI:** 10.3390/ma10060615

**Published:** 2017-06-03

**Authors:** Daisuke Nishiura, Hide Sakaguchi, Akira Aikawa

**Affiliations:** 1 Department of Mathematical Science and Advanced Technology, Japan Agency for Marine-Earth Science and Technology, Kanagawa 236-0001, Japan; sakaguchih@jamstec.go.jp; 2 Railway Dynamics Division, Railway Technical Research Institute, Tokyo 185-8540, Japan; aikawa.akira.11@rtri.or.jp

**Keywords:** discrete element method, viscoelastic deformation, finite element method

## Abstract

Simulation of a large number of deformable bodies is often difficult because complex high-level modeling is required to address both multi-body contact and viscoelastic deformation. This necessitates the combined use of a discrete element method (DEM) and a finite element method (FEM). In this study, a quadruple discrete element method (QDEM) was developed for dynamic analysis of viscoelastic materials using a simpler algorithm compared to the standard FEM. QDEM easily incorporates the contact algorithm used in DEM. As the first step toward multi-body simulation, the fundamental performance of QDEM was investigated for viscoelastic analysis. The amplitude and frequency of cantilever elastic vibration were nearly equal to those obtained by the standard FEM. A comparison of creep recovery tests with an analytical solution showed good agreement between them. In addition, good correlation between the attenuation degree and the real physical viscosity was confirmed for viscoelastic vibration analysis. Therefore, the high accuracy of QDEM in the fundamental analysis of infinitesimal viscoelastic deformations was verified. Finally, the impact response of a ballast and sleeper under cyclic loading on a railway track was analyzed using QDEM as an application of deformable multi-body dynamics. The results showed that the vibration of the ballasted track was qualitatively in good agreement with the actual measurements. Moreover, the ballast layer with high friction reduced the ballasted track deterioration. This study suggests that QDEM, as an alternative to DEM and FEM, can provide deeper insights into the contact dynamics of a large number of deformable bodies.

## 1. Introduction

The simulation of continuum mechanics plays an important role in structural analysis in the fields of coastal engineering and mechanical engineering. In particular, several studies have been conducted using a continuum simulation method, such as the finite element method (FEM), to investigate instantaneous phenomena due to impulsive force, e.g., collision safety performance of automobiles [1], vibration characteristics of railway tracks under traffic impact loads [2,3] and earthquake resistance of buildings and nuclear facilities [4,5]. Although implicit FEM is often used in static or quasi-static problems and modal analysis, its computational cost and stability are affected by the degradation of numerical convergence, which leads to significant issues, especially in dynamic problems, such as collision impact propagation. On the other hand, explicit FEM involves low computational cost per time step, as well as low memory consumption; therefore, it is useful for relatively short-time transient dynamics and large-scale high-performance computing (HPC).

Other dynamic problems, such as fracture and fragmentation processes, transcend the category of continuum mechanics. Thus, in general, it is difficult for FEM to handle the transition process from continuous to discontinuous behavior. Distinct element analysis, using discontinuous deformation analysis (DDA) [6,7], the rigid body spring model (RBSM) [8] and UDEC^®^/3DEC^®^ [9], has been adopted to address discontinuous problems in landslide and avalanche studies. In these methods, artificial joints connected by additional elastic springs are applied between the blocks or elements to handle the transition into discontinuous behavior. In addition, the discrete element method (DEM) [10] can model a structure by connecting particles together similar to artificial joints [11,12,13,14]. Accordingly, deformation and fracture are explained by the stretching and disjointing of the springs. Nevertheless, it is nearly impossible to reproduce the real mechanical properties of continuum material, specifically by using only the spring constant between two particles, because the result obtained by the connecting particle model depends not only on the spring constant, but also on the structural properties of the particle assembly, such as the void ratio, coordination number and particle size distribution. Thus, the constitutive laws of the particle assembly have been reported for limited cases [15]. Consequently, adjustment of the spring constant by trial and error is usually necessary to reproduce the actual phenomena.

To overcome the above problems, the combined finite discrete element method (FDEM) was developed by Munjiza in the early 1990s [16]. FDEM is an effective solution for multi-body dynamics involving viscoelastic deformation and fracture of materials. It combines the advantages of the continuum-based mechanical model and DEM. Numerous discrete elements can be calculated by applying highly efficient methods, such as the strain-based cohesive crack model and the Munjiza no-binary-search (Munjiza-NBS) contact detection algorithm [17], to fragmented elements. Thus, FDEM has been widely applied in the fields of geoscience and geo-engineering [18,19]. For understanding the failure mechanism of composite materials such as concrete, several lattice-based mesoscale models have been developed [20,21,22,23,24,25], which simulate the damage evolution and crack propagation considering the effect of material heterogeneity. The rigid body spring network (RBSN) approach [22] is a promising method for fracture analysis, which is based on the improved RBSM approach by Bolander et al. The lattice discrete particle model (LDPM) [24,25] is also an effective simulation method, which simulates concrete at the mesoscale level by considering the spatial distributions for the size of coarse aggregate particles and the type of materials. In this method, particle contact interaction among adjacent aggregate particles through the embedding mortar is governed by mesoscale constitutive equations, which represents mesoscale tensile fracturing with strain-softening, cohesive and frictional shearing and nonlinear compressive behavior with strain-hardening.

To address the problem of continuum mechanics involving a multi-material and multi-rheology approach, Sakaguchi developed the quadruple discrete element method (QDEM) in the early 2000s [26] with the objective of simulating the three-dimensional interior dynamics of the solid Earth without using the complex modeling techniques of multi-physics. In this field, realistic simulations of the tectonic process in the Earth, including mantle convection, plate subduction and magma flow, require the consideration of a wide variety of geomaterials with complex rheology. Thus, it is necessary to address different mechanical properties and interactions among different geomaterials, which is favorable for DEM, but not for FEM. However, it is difficult for DEM to reproduce the mechanical properties of the continuum body, as mentioned above. Therefore, it is necessary to develop a new DEM-like method to exactly reproduce the continuum behavior without the iterative solution of the simultaneous equations in FEM.

In DEM, the inter-particle constitutive relationship is always a one-dimensional model between two particles, even for a two- or three-dimensional problem. The one-dimensional model cannot precisely express the changes in surface area and volume. At the least, a constitutive law of the assembly of four particles (quadruple discrete element) is required for volume changes in consideration of a three-dimensional simulation. Thus, in accounting for the computational cost, interactive force (stress) is defined by QDEM as the minimum number of particles. The viscoelastic stress is explicitly calculated in an element-by-element manner on the basis of a constitutive law that employs the real viscoelastic parameters of the materials. The particles are then individually moved according to their interactions with the surrounding elements. As a result, the computational scheme of QDEM is nearly the same as that of FDEM in terms of the continuum dynamics.

Nevertheless, QDEM and FDEM are based on different concepts, as mentioned above. Furthermore, QDEM cannot handle fracture and fragmentation processes because its focus is high-performance computing for seismic wave propagation in the solid Earth interior [27,28]. In the simulation of seismic wave propagation, it is required to address the different viscoelasticities of the geomaterials at a high spatial resolution. Thus, the computational scheme should be developed using a model that is as simple as possible in order to reduce the computational cost.

QDEM is a simple algorithm that consumes minimal memory and exhibits high computational efficiency because it does not require the calculation of global matrices as in the case of FEM. In addition, the QDEM algorithm is suitable for large-scale parallel computing. By contrast, FEM, especially implicit FEM, requires a special technique to realize scalable parallel performance because the constitutive equation must be solved using global matrices, such as mass, stiffness and damping matrices. In explicit FEM, some of these global matrices can be diagonalized as lumped matrices to improve parallel performance. However, the global stiffness matrix cannot be diagonalized. Thus, the communication cost of parallel computing remains high. Furthermore, when considering damping, the simultaneous equations are generally required to be solved iteratively, even if the solution is an explicit method. This is because the global damping matrix can be diagonalized only in the special case of using Rayleigh damping combined with modal analysis. Nevertheless, the relationship between the actual physical viscosity and the virtual viscosity in Rayleigh damping remains ambiguous. Therefore, the damping parameters have to be determined either by fitting the experimental or theoretical values or by relying on experience.

In contrast to FEM, QDEM can easily implement high-efficiency parallelization without calculating the global matrices because it not only calculates the stress in an element-by-element manner, but also calculates individual particle motion using only local matrices with the components of surrounding elements sharing a particle. The parallel performance of QDEM can be further improved by adopting a recent dynamic load balancing technique [29,30] because the stress calculation is localized. In particular, the QDEM algorithm is compatible with shared memory parallelization techniques for particle simulation [31,32]. It can effectively leverage a variety of current hardware with an increasing number of arithmetic processor cores and threads, such as the many integrated core (MIC) processor and graphics processing unit (GPU), which have been widely used in recent years. Although we additionally implemented multi-GPU computing in this study, the details of its implementation are omitted herein because it is not relevant to our present objective.

As mentioned above, the parallel performance of QDEM is expected to be superior to that of FEM because QDEM does not require the calculation of global matrices. Nevertheless, it is important to determine whether a theoretical solution can be reproduced by QDEM without computing global matrices or shape functions. If QDEM can reproduce a theoretical solution, it could replace FEM. On the other hand, FDEM is rarely used as an alternative to FEM for continuum analysis; instead, it is often used in multi-body dynamics and fracture analysis. Its applicability to continuum analysis, especially of transient processes, remains unknown owing to a lack of information on its accuracy when applied to a continuous problem, because the principal objective is the solution of a discontinuous problem. If the computational accuracy of QDEM, as well as that of FDEM, is comparable to that of FEM, then QDEM and FDEM may be deemed more useful than FEM because of their simpler algorithms and easier implementation on HPC systems.

Although we recognize the advantage of FDEM in handling fracture dynamics, we believe that it is important to verify the computational accuracy of both QDEM and FDEM in structural analysis without fracture in order to elucidate their usefulness as alternatives to FEM. Therefore, in this study, we verify the reproducibility of the basic behavior of viscoelastic materials by QDEM. The displacement and natural frequency of elasticity and the transient damping of viscosity are compared with FEM and the analytical solution, respectively. Consequently, through the verification of viscoelastic behavior, we report the information that is necessary for determining whether the continuum simulation method without the calculation of matrices can be an alternative to FEM.

The final objective of this study is to simulate and analyze contact dynamics for a large-number of viscoelastic deformable bodies by applying the above-mentioned reliable QDEM. As a suitable example, QDEM was applied to the impact response analysis of ballasted railway tracks for cyclic loading induced by a passing train. The dynamic loads of the trains are transmitted to the ballasted layer as an elastic wave through the inside of the ballast particles, and they consequently induce the natural vibration modes in the ballast layer, which trigger deterioration of the ballasted track. Therefore, the ballasted track requires periodic maintenance, which is an important consideration for railway management in terms of reducing the maintenance cost. However, the dynamic characteristics of the ballast layer have not been sufficiently elucidated thus far. To gain deeper insights into the causes of ballasted track deterioration, it is necessary to develop an advanced method to simulate the dynamic internal behavior of the ballast layer with high precision. In this study, first, we apply the interaction force calculation used in DEM to QDEM in order to deal with the frictional collision among objects, such as ballast-to-ballast and ballast-to-sleeper interactions. Then, we simulate the ballasted track behavior influenced by actual traffic impact load associated with a passing train. Further, we investigate the frictional effect of the ballast layer on track deterioration due to the elastic vibration and displacement of the ballast particle. Finally, we compare the vibration characteristics of the ballast and sleeper with actual field-measured values obtained using a special sensing ballast particle and sensing sleeper [33] in order to confirm the reliability of our simulation method.

## 2. QDEM Concept and Implementation

**Figure 1:**
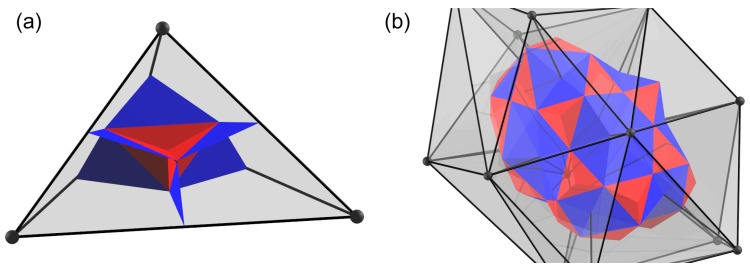
(**a**) Triangular surfaces subdividing the tetrahedron into four subdomains with equal volumes by using the tetrahedron centroids. The blue region consists of two centroids of the surface and one centroid of the edge, and the red region consists of two centroids of the surface and one centroid of the volume. (**b**) One volume element enclosed by the subdivided triangular surfaces.

In general particle models, interactive force is considered between two particles regardless of the dimension in space. For example, in ordinary DEM, elastic force is determined as a function of the relative displacement of two particles in contact. In the simplest case, such as that of a linear elastic model, a spring constant characterizes the interactive force. When many particles in contact are bonded with springs and form a one-dimensional bar, the bulk behavior exactly shows the one-dimensional linear elasticity characterized by the spring constant. By contrast, if the bonded particles with springs form a tetrahedron, the bulk behavior does not match the real three-dimensional linear elasticity, as mentioned in Section 1. Therefore, to overcome this problem, instead of a two-particle interaction model, a four-particle interaction model is introduced as an extension of the particle model. This simple concept is the basis of QDEM. In terms of the original problem of describing the mechanical behavior of continuum materials by particle modeling, we must determine the type of space discretization that should be adopted in QDEM. Under the QDEM concept, a tetrahedron is not a representation of one finite element as in FEM; instead, it is a representation of the interacting part of four particles. Thus, we must decompose the tetrahedron into four equal-volume subdomains to attribute the interactive force to each particle, as shown in Figure 1a. For an arbitrarily-shaped tetrahedron, four equal-volume subdomains can be defined by the following three processes.

1. Find one volume centroid $vc=vc_{1234}$, four face centroids $\left\{fc_{123},fc_{124},fc_{134},fc_{234}\right\}$ and six edge centroids $\left\{ec_{12},ec_{13},ec_{14},ec_{23},ec_{24},ec_{34}\right\}$ in the tetrahedron. The subscripts denote the vertex indices.
2. Find six triangles composed of one edge centroid and two face centroids $\{<ec_{12},fc_{123},fc_{124}>$, $<ec_{13},fc_{123},fc_{134}>$, $<ec_{14},fc_{124},fc_{134}>$, $<ec_{23},fc_{123},fc_{234}>$, $<ec_{24},fc_{124},fc_{234}>$, $<ec_{34},fc_{134},fc_{234}>\}$. In addition, find six triangles composed of the volume centroid and two face centroids $\{<vc,fc_{123},fc_{124}>$, $<vc,fc_{123},fc_{134}>$, $<vc,fc_{123},fc_{234}>$, $<vc,fc_{124},fc_{134}>$, $<vc,fc_{124},fc_{234}>$, $<vc,fc_{134},fc_{234}>\}$.
3. Decompose the tetrahedron with the 12 triangular surfaces defined by Process 2. Each decomposed subdomain represents a part of each particle that contributes to the four-particle interaction.

If we assume that a tetrahedron vertex is a representative point of a particle, the total volume of the particle can be defined by the space filled with all of the subdomains of the tetrahedron that share the vertex. Thus, each particle exhibits a potato-like shape, as shown in Figure 1b. Consequently, the three-dimensional space is filled with the potato-like particles, and the interactive force is computed among the interfaces of the four adjacent particles belonging to the tetrahedron [34]. The force is distributed to each particle. Then, the particle motion is calculated according to Newton’s equation of motion. Based on this concept, the details of the viscoelastic model and its implementation for QDEM are provided in the following subsections.

### 2.1. Elastic Model with Hooke’s Law

To calculate the deformation in QDEM, we must define the elastic stress for the tetrahedron, as shown in Figure 1a. Let us consider isotropic linear elastic materials, whose Cauchy stress only linearly depends on the deformation gradient. The deformation gradient is defined by the relationship between the material line elements in the reference configuration, dX, and the current configuration, dx, as follows:

(1) $$dx=FdX\;\;\;\;\;∴F=dxdX^{-1}\;.$$

the second-rank tensor, F, is the deformation gradient, which maps the vectors from the reference configuration onto vectors in the current configuration in the three-dimensional space. Hence, it is also known as a two-point-tensor. Thus, to determine the nine components of F, at least three vectors in a linearly independent set are required. If four points that are not arranged on the same plane are given, we can obtain the three linearly independent vectors. This means that the components of dX and dx consist of the three vectors from any point to the other three points. In addition, because the four points form a tetrahedron, we can define F for the tetrahedron by tracing the positions of those four points from the reference configuration.

Here, let us consider an isotropic elastic body obeying Hooke’s law for small deformations. Hence, the Cauchy stress tensor is given by:

(2) $$T_{E}=\lambda_{E}\mathrm{tr}\left(\epsilon \right)I+2\mu_{E}\epsilon \;,$$

where $\lambda_{E}$ and $\mu_{E}$ are the first and second Lamé constants, respectively, ϵ is the linearized (infinitesimal) strain tensor and I is the second-rank identity tensor. We choose the left Green–Lagrange strain tensor, ϵ=(B−I)/2, as the strain tensor by using the left Cauchy–Green deformation tensor $B={\mathrm{FF}}^{T}$. Here, F can be decomposed into two parts by polar decomposition as:

(3) F=VR.

thus, B is

(4) $$B={\mathrm{FF}}^{T}=V^{2}\;\;\;\;\;∵{\mathrm{RR}}^{T}=I,V=V^{T}\;,$$

where R is an orthogonal tensor corresponding to a rigid rotation and V is a positive definite symmetric left stretch tensor corresponding to a pure strain. Unlike the right Cauchy–Green deformation tensor, the strain can be obtained from the reference and current configurations visualized in the same global (initially fixed) coordinate system because the stretch V is measured after rotation in the case of B. Therefore, by tracing only four vertexes of the tetrahedron in a global coordinate system, we can simply calculate the elastic stress from the strain through the deformation gradient.

### 2.2. Viscoelastic Model for Kelvin–Voigt Materials

We consider the viscous stress in the tetrahedron, which linearly depends on the strain rate corresponding to the velocity gradient. For viscous materials, the dependency of the deformation gradient of elastic materials on the Cauchy stress is replaced by the dependency of the velocity gradient. The velocity gradient is defined by the relationship between the velocity dv and the position dx of the material line elements in the current configuration as:

(5) $$dv=Ldx\;\;\;\;\;∴L=dvdx^{-1}\;.$$

the second-rank tensor, L, is the spatial velocity gradient. Here, dv is the time differential of dx, which can be obtained from the velocities of the four vertexes of the tetrahedron. The velocity gradient tensor also consists of stretching and rotating parts, as well as the deformation gradient F. Thus, L can be uniquely decomposed into the symmetric part, D, and the anti-symmetric part, W, as:

(6) L=D+W,

where $D=(L+L^{T})/2$ and $W=(L-L^{T})/2$ are called the strain rate tensor and the spin tensor, respectively. If we assume isotropic linear viscous materials, the Cauchy stress depends only on the symmetric part D of L by the following representative form in the same manner as in Equation (2).

(7) $$T_{D}=\lambda_{D}\mathrm{tr}\left(D\right)I+2\mu_{D}D,$$

here, $\mu_{D}$ is the shear viscosity, and $\lambda_{D}+2/3\mu_{D}$ is the bulk viscosity. On the other hand, for the viscoelasticity of Kelvin–Voigt materials, the Cauchy stress can be represented by:

(8) $$T=T_{E}+T_{D}\;,$$

which means that the viscous and elastic terms are connected in parallel. Therefore, by only tracing the four vertexes of the tetrahedron, we can simply obtain the viscoelastic stress by calculating the viscous stress from the strain rate through the velocity gradient along with the elastic stress, as mentioned earlier.

### 2.3. From Tetrahedron Stress Tensor to Particle Force Vector

To calculate particle motion, we must obtain the force vector acting on the particle from the tetrahedron stress tensor given by Equation (8). In QDEM, if we assume that the stress distribution in the tetrahedron volume is homogeneous, the stress tensor may be converted into the force vector acting on the volume element, which is constructed by the surrounding triangular surfaces around each particle, as shown in Figure 1b. Stress vector $t_{i}$ on the *i*-th triangular surface with unit normal vector $n_{i}$ is given by stress tensor T according to $t={\mathrm{Tn}}_{i}$ of Cauchy’s formula. It is then converted into force vector $f_{i}$ acting on surface area $S_{i}$ as:

(9) $$f_{i}=t_{i}S_{i}=\frac{1}{2}T\left(a_{i}\times b_{i}\right)\;.$$

here, a and b are the edge vectors of the triangle, S=|a×b|/2 and n=a×b/|a×b|, whose direction is from the triangle surface to the corresponding tetrahedron vertex. All force vectors corresponding to subset *A* within the entire set *B* of triangular surfaces are summed in each subdomain. Force vector ${\tilde{f}}_{j}$ on the particle belonging to the *j*-th subdomain is given by:

(10) $${\tilde{f}}_{j}=\sum_{i\in A_{j}\subset B}f_{i}\;\;\;\;\;j\in \left\{1,2,3,4\right\}\;.$$

The implementation described above is based on the unique idea that each particle has a volume enclosed by many subdivided triangular surfaces in the surrounding tetrahedrons, and its volume element receives its surface forces, f. However, more simply, an equivalent result may also be obtained to equally distribute the force defined on the triangular surface of the tetrahedron to each particle.

### 2.4. Material Properties and Computational Procedure

In the previous subsections, we described the calculation method of isotropic linear viscoelastic stress for a tetrahedron. To simulate real viscoelastic materials, we must represent the geometry of the material body by using multiple tetrahedrons. Therefore, in reality, the proposed method is applied for each tetrahedral element, and it explicitly calculates the element stress of Equation (8). Then, the particle forces of Equation (10) are calculated for each element and are summed on the shared particle by the surrounding elements (*j*-th subdomains) as the *i*-th equivalent particle force,

(11) $$p_{i}=\sum_{j\in C_{i}\subset D}{\tilde{f}}_{j}\;\;\;\;\;i\in N\;,$$

where *N* is the set of all particle indices and $C_{i}$ is the set of surrounding subdomain indices around the *i*-th particle in the entire set, *D*, of subdomains.

Similarly, the fourth part of the tetrahedron mass should be distributed to each particle. It is summed on the shared particle as an equivalent particle mass because the stress is distributed to the subdomains with equal volumes, as mentioned above. According to particle force p and the mass, the particle motion is calculated by the explicit Verlet time integration scheme of Newton’s equation of motion. Note that the material properties are assumed to be uniformly distributed in homogeneous materials for an infinitesimal deformation. Thus, the material properties of each element, such as the viscosity, density and Lamé constants determined by the relationship between the Young’s modulus and the Poisson’s ratio, are set to the same values over the entire material body.

## 3. Verification Tests

### 3.1. Elastic Body

**Figure 2:**
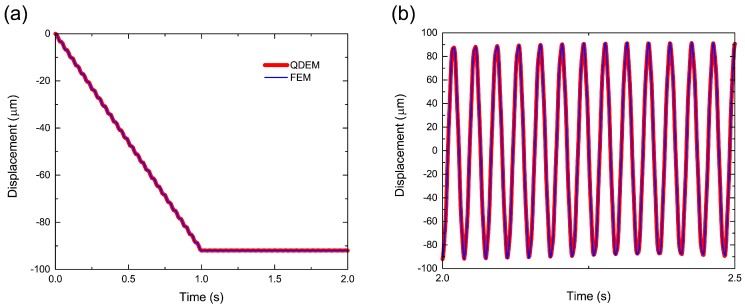
Time variations of displacement at the center of the cantilever free end (**a**) under loading and (**b**) after unloading.

We applied QDEM to three-dimensional deformation analysis of a cantilever beam. The Young’s modulus, Poisson’s ratio and density were set to 45 GPa, 0.167 and 2350 kg/m^3^, respectively, as the material properties of concrete. For elastic analysis, the viscosity was set to zero. The length, width, and height of the cantilever were 2.4 m, 0.25 m and 0.22 m in the *x*-, *y*- and *z*-directions, respectively. The cantilever was decomposed into 488,361 tetrahedral elements and 94,149 particles, as shown in Figure S1 of the Supplementary Material. One side of the length was the fixed end; the other side was the free end. The total downward load in the *z*-direction was 200 N (3.6 kPa), and it was applied to all particles in the free end. Here, the load was linearly increased from 0 N to 200 N in 1 s to avoid oscillation in the static state. After 1 s, the displacement was steady, and its average and standard deviations over 1 to 2 s were 91.94045 μm and 0.0550 μm, respectively, at the center particle of the free end, as shown in Figure 2a.

The time variation of the displacement was fit to the curve of implicit finite element (FE) analysis using FrontISTR [35], which uses the Newmark-β (average acceleration) method for time integration. Moreover, it uses a conjugate gradient (CG) solver for simultaneous equations. In the FE analysis, the average steady displacement was 91.93975 μm, which was 0.0008% smaller than that obtained by QDEM. Note that the primary tetrahedral element used in FE analysis was the same as that used in QDEM. In addition, the displacement obtained by the analytical solution of the uniaxial model (Equation (12)) was 92.32156 μm. Thus, the relative error between the analytical solution and QDEM was 0.4%.

(12) $$\delta =\frac{P_{z}L_{x}^{3}}{3EI_{yz}},$$

**Figure 3:**
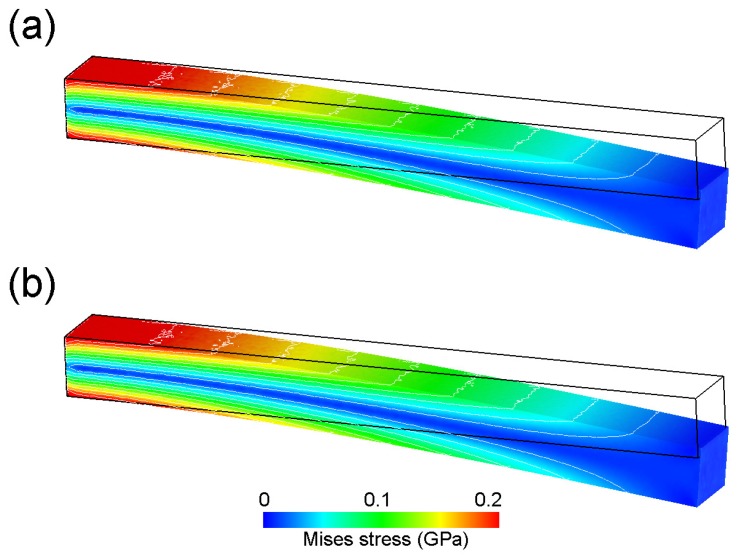
Spatial distribution of von Mises stress. The results of (**a**) QDEM and (**b**) FEM show the stress in the steady state at 2 s after loading began. The displacement is shown with a magnification of 2000×.

Here, *P* is the load, and *E*, *I* and *L* are Young’s modulus, the second moment of the area and the cantilever length, respectively. The subscripts denote the dimensional directions. Figure 3 shows the spatial distribution of the von Mises stress at 2 s. Thus, QDEM could perfectly reproduce the stress distribution of FE analysis within 0.04% of the average relative error (the maximum error was 0.12%) in an element-by-element manner, as well as in the time history of the displacement.

To analyze the dynamic behavior of the cantilever, we unloaded the downward load after applying the load at the free end for 2 s. Figure 2b shows the time variation of the displacement at the center particle of the free end after unloading. The frequency and amplitude of the vibration in QDEM were in good agreement with those in the FE analysis. The frequency was 26.98 Hz obtained by fast Fourier transform (FFT) analysis, which was quantitatively equal to the first natural frequency (27.01 Hz) of the analytical solution given by:

(13) $$f=\frac{1.875^{2}}{2\pi L_{x}^{2}}\sqrt{\frac{EI_{yz}}{\rho \alpha_{yz}}}\;,$$

where α is the cross-section area and ρ is the density. The relative error between the QDEM simulation and the analytical solution was 0.1%. Thus, for static and dynamic analysis of elastic materials, it was clarified that the accuracy of QDEM is the same as that of FEM using the primary element. Note that the total computational time was approximately two days using a discrete time step of 10^−7^ s with one GPU card (Nvidia Co., GeForce GTX Titan). FEM required one day using a discrete time step of $3\times 10^{-5}$ with 16 CPUs (Intel Co., Core i7-5960X). In the case of FEM, the CPU occupancy rate was practically 3% to 4% of the total computational time because the memory access cost exceeded the arithmetic cost for solving the massive matrices. Therefore, the QDEM computational speed per step was approximately 300-times higher than that of FEM.

### 3.2. Viscoelastic Body

To investigate the effect of viscosity, we analyzed the two motion types in the quasi-static and dynamic regimes by using the same geometry as that described in Section 3.1. The density was set to 2350 kg/m^3^, while the Young’s modulus and the time step were set to 45 GPa and 10^−8^ s for the dynamic regime and 0.5 MPa and 10^−7^ s for the quasi-static regime, respectively. For simple comparison with analytical solutions, the bulk viscosity and Poisson’s ratio were set to zero in this analysis. As the boundary condition, the bottom side of the bar was the fixed end. On the other hand, the top side was fixed in the *x*- and *y*-directions, while it was free to move in the *z*-direction. Initially, the total *z*-direction downward load was 10 N (16.6 Pa), and it was continuously applied to all of the particles in the top side.

**Figure 4:**
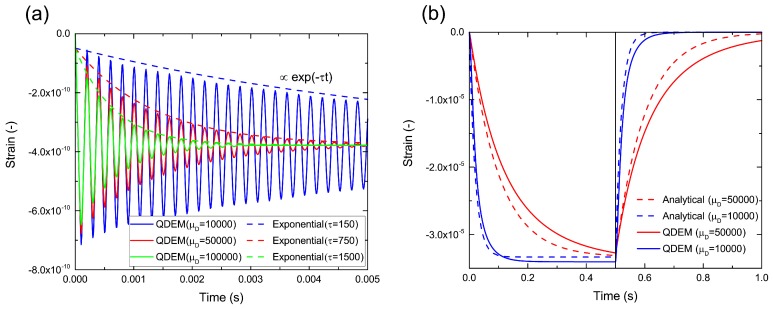
Time variations of strain at the center of the top side of the bar for the (**a**) dynamic regime and (**b**) quasi-static regime.

Figure 4 shows the time variation of the strain at the center of the top side for each shear viscosity. For the dynamic regime of the damped vibration shown in Figure 4a, it was confirmed that the attenuation of the strain amplitude increased with the viscosity. Each curve fitted to the amplitude peaks is represented by the natural exponential function of $3.29\times 10^{-10}\exp\left(-\tau t\right)-3.78\times 10^{-10}$. The ratio between the reciprocal relaxation time τ and the corresponding viscosity $\mu_{D}$ was a constant value of 0.015 under different conditions. On the other hand, for the quasi-static regime of over-damping, shown in Figure 4b, we observed the creep phase and recovery phase after unloading at $t_{0}=0.5$ s, which is a typical feature of the Kelvin–Voigt model. In comparison with analytical solutions given by:

(14) ε=PzEαxy1−e−tEtEμμDt≤t0PzEαxye−tEtEμμDe−t0Et0EμDμD−1t≥t0,

the response of the strain change in QDEM was slightly slower. We believe that the delay may be primarily attributed to the boundary condition. In this study, the top and bottom ends were fixed (nonslip) to avoid elastic oscillation due to the inertial effect. The fixed boundary condition restricted the deformation flexibility of the bar, causing apparent hardness.

If we examine the creep-recovery test over a long period, the inertial effect might be negligible, and we could adopt the slip boundary. However, it would be nearly impossible to simulate the long-term evolution using an explicit method with a fine time step owing to the computational cost. Both static and quasi-static problems should typically be solved by an implicit method [36,37], regardless of whether the inertia properties are considered. Consequently, we believe that QDEM can effectively reproduce the effect of viscosity on the viscoelastic behavior within the region, which can be handled by the explicit method. In addition, QDEM can represent the damping effect by using only the scalar parameter of the real viscosity coefficient without the virtual (Rayleigh) damping matrix, which is proportional to mass and/or stiffness using FEM.

## 4. Ballasted Track Simulation

In this study, our simulation target is the behavior of the ballast layer and sleeper. First, we implemented the frictional contact force calculation in QDEM to deal with the ballast-to-ballast and ballast-to-sleeper interactions. For simplicity, we used the contact detection algorithm in DEM, which offers a significant advantage in terms of the computational cost. Moreover, the existing parallel computing algorithm, which was developed by the author in previous studies for shared-memory parallel computing of DEM [31,32], can be used as is. Here, a spherical particle having a size of 24 mm was installed at each vertex of the tetrahedral element of the ballast and sleeper. This particle was used only for contact detection and contact force calculation. For reference, because the averaged edge length of the tetrahedral element was 10 mm, the neighboring particles were overlapped with each other such that the overlap region was larger than half of the particle size on average. Thus, the object had a relatively smooth surface because of the overlap although the installed particles influenced the object shape. The contact force was estimated by the Voigt model with a frictional slider, but the damping term due to the dashpot was not considered because the viscous damping effect has already been considered in the constitutive equation (Equation (7)) of QDEM.

For the setup of the real simulation model, a Type-3 pre-stressed concrete (PC) mono-block sleeper was used, which is widely employed in conventional railway lines of the Japan Railway (JR) companies. The maximal length, width and height were 2 m, 0.24 m and 0.174 m, respectively; the other properties of the Type-3 PC sleeper have been described by Sakai and Aikawa [33]. The sleeper and ballast particle were divided into tetrahedral elements to calculate the viscoelastic deformation by QDEM. The illustration of the actual element used for the sleeper is shown in Figure S2 of the Supplementary Material. All of the ballast particle shapes were different, and they were randomly made by hand. Each ballast particle had approximately 100 tetrahedral elements, and its size was around 5 cm. The physical properties used in the simulation are listed in Table 1, which are the real material parameters of the ballast and sleeper except for the viscosity and friction coefficient. Because the viscosity of a solid and the friction coefficient of irregularly-shaped particles are usually difficult to determine, these parameters were set as realistic values in consideration of previous studies [38] and computational stability.

The simulation system size was one sleeper region, the length, width and height of which were 5.0 m, 0.6 m and 0.7 m, respectively. The periodic boundary condition was imposed in the width direction. Initially, the sleeper was embedded into the ballast layer to 0.1 m from the bottom of the sleeper; then, the initial position of the ballast particles and sleeper was set by free fall. Consequently, the bottom length, top length, width and height of the ballast layer were 4.6 m, 3.0 m, 0.6 m and 0.4 m, respectively.

**Figure 5:**
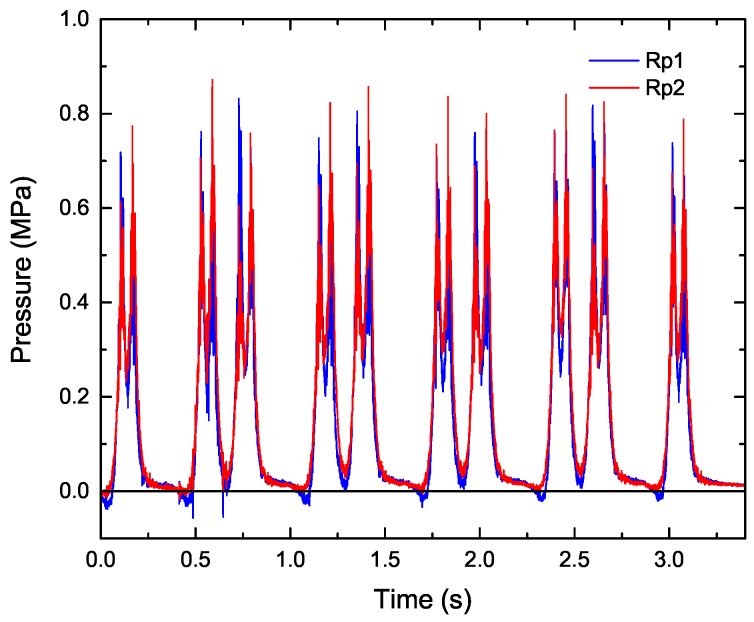
Time history of pressure between sleeper and rail at positions of the right rail (Rp1) and the left rail (Rp2). In these data, the number of passing cars was five, where one car had two bogies, and each bogie had two axles.

**Figure 6:**
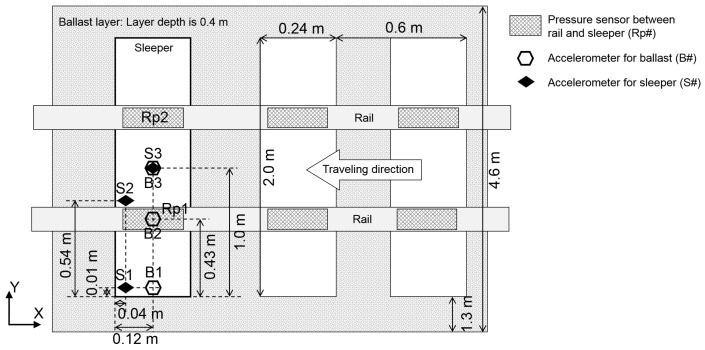
Position diagram for measurements of pressure between the rail and sleeper and accelerations of the ballast particle and sleeper. Accelerometers for the ballast particle were installed in the ballast layer below 10 cm from the bottom of sleeper. Accelerometers for the sleeper were installed on top of the sleeper.

As the input load for ballasted track simulation, we used actual time series data of the pressure, as shown in Figure 5, which were measured between the sleeper and the rail at the positions of the right rail (Rp1) and the left rail (Rp2), as shown in Figure 6. In the simulation, we converted the pressure into the load per vertex particle of tetrahedral elements within the area of the pressure sensor and then uniformly applied the load to the vertex particles. Note that because the sampling rate of the pressure sensor was only 10 kHz, whereas the input rate was 10 MHz owing to a simulation time step of 10^−7^ s, the input data were linearly interpolated between the time series data. To analyze the impact response of the ballast and sleeper, we measured the acceleration and displacement at the same position as that in an actual field measurement, as shown in Figure 6. The acceleration was measured at a sampling rate of 10 kHz in both the simulation and the actual measurement.

### 4.1. Track Deterioration with Cyclic Loading

**Figure 7:**
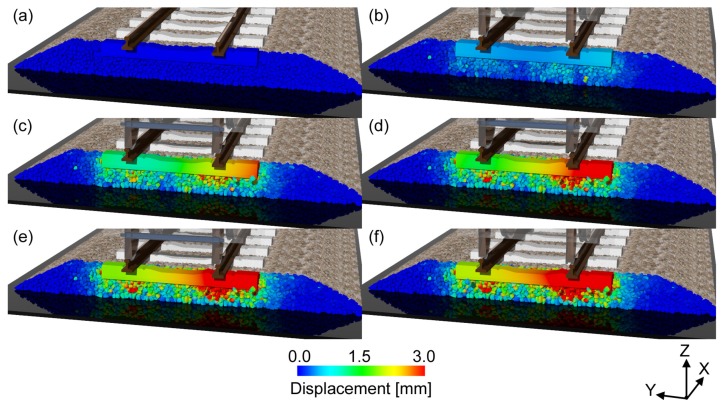
Snapshots of displacement of the ballast particle and sleeper at each traveling time tof the train: (**a**) *t* = 0.0 s, (**b**) *t* = 0.11 s, (**c**) *t* = 0.73 s, (**d**) *t* = 1.35 s, (**e**) *t* = 1.98 s and (**f**) *t* = 2.61 s. The displacement is shown with a magnification of 20×. A video sequence of this simulation can be found as the Supplementary Material in Video S1.

The investigation of the rate of track deterioration is important for managing the maintenance period of the ballasted track. We simulated the behavior of the ballast and sleeper induced by the cyclic loading. Figure 7 (see also Video S1 in the Supplementary Materials) shows the displacement of the ballast and sleeper at each traveling time of the train. To observe the internal view, we have not shown the ballast particles located below 0.2 m in the *x*-direction. Figure 7a corresponds to the initial time, and Figure 7b–f correspond to the times when the front axle of each car reached the sleeper. From these figures, we can observe that the displacement of the ballast particle around the sleeper increases with the passage of the train axle, although both sides of the ballast layer remain stable. In addition, the ballast particle displacement under the rail is larger than that at the center. In particular, the ballast particle displacement under the right rail (Rp1) is larger than that under the left rail (Rp2). Note that the input loads at Rp1 and Rp2 are different. The pressure on the right rail is sometimes negative, as shown in Figure 5, which means that the right side of the sleeper is about to lift up. Therefore, the right side of the sleeper vibrated considerably, and the ballast particles of the right side were then considerably moved by the sleeper vibration.

**Figure 8:**
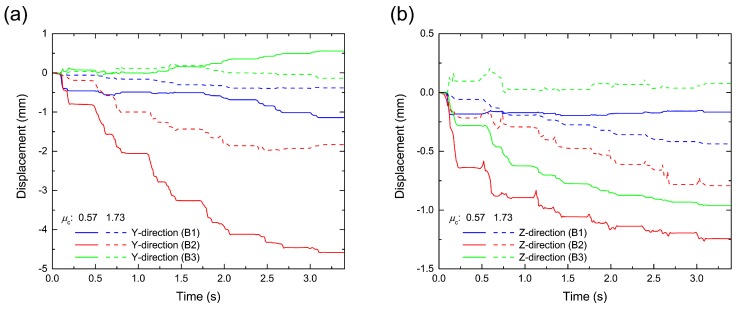
Time variations of displacements in (**a**) the lateral *y*-direction and (**b**) the vertical *z*-direction for each ballast particle located at B1, B2 and B3. The solid and dashed lines represent the results with frictional coefficients ($\mu_{c}$) of 0.57 and 1.73, respectively. These figures correspond to the time history of pressure shown in Figure 5, where the number of passing cars was five.

Next, we investigated the details of the ballast particle motion. Figure 8 shows the time variations of the ballast particle displacement at each position (B1, B2 and B3). First, the ballast particle suddenly settled during the passage of the first car, as shown in Figure 8b, because the ballast layer, which is formed by free fall, was initially not consolidated. Thus, the packing structure of the ballast layer influences the track deterioration. Then, the maximum ballast particle settlement occurred under the rail (B2) compared to the side (B1) and center (B3). It is assumed that these settlements were induced by the associated lateral motion, as shown in Figure 8a, because the lateral motion, which is larger than the vertical motion, makes space for settlement in the ballast layer. Moreover, we confirmed that the settlement and lateral motion decrease as the frictional coefficient $\mu_{c}$ of the ballast particle increases from 0.57 to 1.73. Therefore, friction has a significant influence on track deterioration. In practice, the friction depends on the ballast particle shape properties, such as surface roughness and edge sharpness. Thus, to reduce the ballasted track deterioration, it is important to control not only the packing structure of the ballast layer, but also the ballast particle shape due to crushing.

### 4.2. Impact Response Analysis of the Ballast and Sleeper

**Figure 9:**
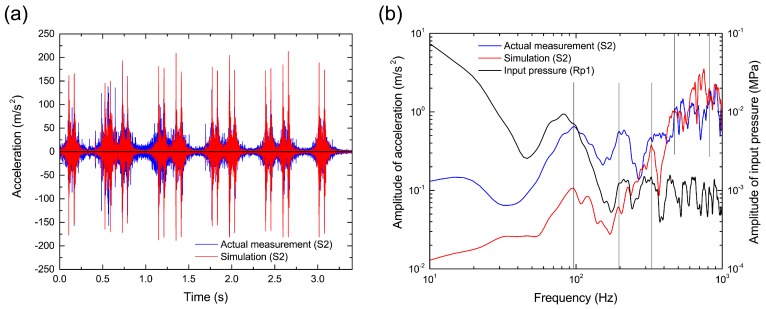
Comparison between numerical simulation and actual measurement in terms of vibration properties of sleeper at sensor position S2. (**a**) Time variation of sleeper acceleration in the vertical direction and (**b**) linear spectra of the sleeper acceleration. For reference, the linear spectrum of the input pressure loading, as shown in Figure 5, is also shown in (b). The linear spectra are smoothed by applying a Parzen window with a bandwidth of 20 Hz.

In Section 4.1, we confirmed that the ballast particle settlement is associated with the sleeper vibration. Then, we analyzed the property of the sleeper vibration using fast Fourier transform (FFT) analysis. Figure 9a shows the time variation of sleeper acceleration near a rail (S2) during the passage of five cars. We confirmed that the acceleration increased sharply when each train axle reached the sleeper, as observed in the actual measurement. To illustrate the details of the acceleration changes, Figure 9b shows the linear spectrum obtained by FFT analysis for the time history of the vertical acceleration during the stable period of ballast particle settlement from 2.85 s to 3.26 s, as shown in Figure 8. In addition, we decided to focus on the results below 1 kHz as reliable values because the sampling rate of the measurement was 10 kHz. The peak positions of the simulation were similar to those of the actual measurement because the same pressure load as the actual measurement was applied on the sleeper in the simulation. However, the amplitude was different from the actual measurement because of differences in the ballast particle configuration such as the packing structure, particle size and particle shape between the simulation and actual measurement. Figure 9b also shows the linear spectrum of the input pressure loading for reference. Note that the frequency of sleeper vibration influences the linear spectrum of the input load because a pressure sensor is installed between the rail and the sleeper to measure the input load. In general, the sleeper receives an input load at low frequency (up to several tens of Hertz) that is derived by applying a heavy wheel load on the rail during the passage of a train at high speed. The sleeper receives an input load at high frequency (from a few hundred up to a million Hertz) that is applied by the rolling contact mechanism of the wheel on the rail. Under these input loading conditions, the sleeper acceleration spectra in the simulation and actual measurement have similar peaks around 100 Hz, which is known to be characteristic of a rigid body (non-bending) vibration in the vertical direction [39]. We also confirmed other peaks around 200 Hz, 400 Hz and 800 Hz, which are related with the first, second and third vertical bending modes, respectively, obtained from natural frequency (modal) analysis of the Type-3 PC sleeper [33]. We also confirmed other peaks at around 200 Hz, 400 Hz and 800 Hz, which are related to the first, second and third vertical bending modes, respectively, obtained from the modal analysis; however, a peak is not observed at around 320 Hz in the modal analysis because the sleeper motion is assumed to be a resonance mode with elastic vibration due to the stretching motion of the ballast layer, which exists at around 320 Hz [40]. Therefore, we first reproduced the sleeper vibration induced by the elastic vibration of the ballast layer by coupled simulation of the sleeper and ballast particles.

**Figure 10:**
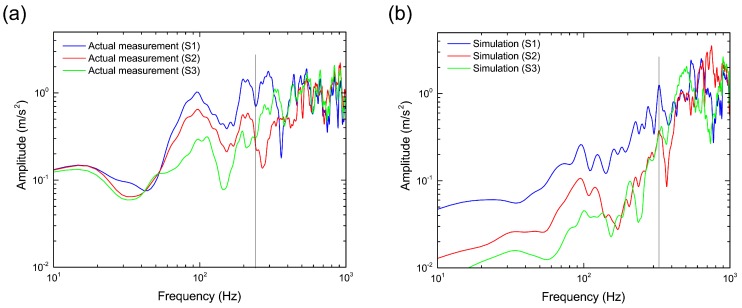
Comparison of frequencies of sleeper vertical acceleration at different sensor positions in (**a**) actual measurement and (**b**) numerical simulation. The linear spectra are smoothed by applying a Parzen window with a bandwidth of 20 Hz.

The frequencies of the other sensor positions are also shown in Figure 10. The acceleration amplitude of the actual measurement increases toward the sleeper side S1 from the center S3 in the frequency region below around 250 Hz. Above 250 Hz, the amplitude of S3 becomes larger than that of S2. In the simulation, the boundary frequency was confirmed to be 320 Hz where the magnitude relation of the acceleration amplitude is reversed as seen in the actual measurement. Thus, although a difference of 70 Hz in the boundary frequency was observed between the simulation and actual measurement, QDEM was able to qualitatively reproduce the vibration characteristics of the sleeper in the ballasted track induced by the traffic impact load.

**Figure 11:**
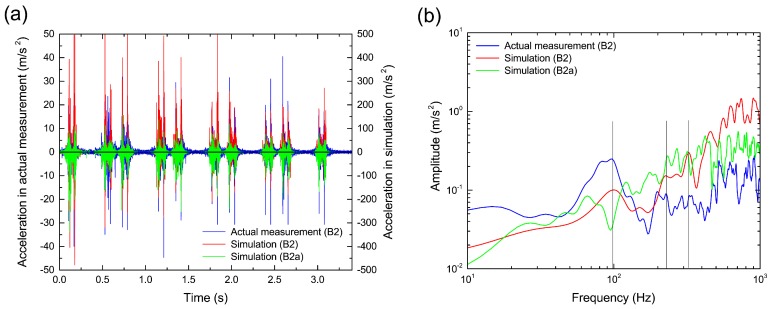
Comparison between numerical simulation and actual measurement in terms of vibration properties of ballast particle. (**a**) Time variation of ballast particle acceleration in vertical direction and (**b**) linear spectra of ballast particle acceleration. The linear spectra are smoothed by applying a Parzen window with a bandwidth of 20 Hz. The red and blue lines denote the ballast particle at sensor position B2. The green line denotes one of the neighboring ballast particles around sensor position B2.

On the other hand, for the ballast particle at position B2 under the rail, the time variation of ballast particle acceleration is shown in Figure 11a. For reference, the result of the neighboring ballast particle around B2 is also shown as B2a. Although the timings of the acceleration peaks in the simulation and actual measurement are identical, the acceleration magnitude in the simulation is up to 10-times larger than that in the actual measurement. In addition, by comparing B2 and B2a, we confirmed that there are individual differences in the ballast particle motion that depend on the condition of the ballast particle arrangement, such as the contact configuration between ballast particles and rotation (orientation) of the irregularly-shaped ballast particles. Thus, we concluded that the magnitude difference in ballast particle acceleration occurred because it was impossible to construct the same structure of the ballast layer as that used in the actual measurement. However, the frequency of the acceleration can be roughly reproduced as shown in Figure 11b, which is for the vertical acceleration time history from 2.8 s to 3.2 s. In particular, we believe that the peaks around 100 Hz, 200 Hz, 400 Hz and 800 Hz depend on the sleeper vibration because these peaks were observed at the same frequencies as those of the natural vibration mode of the sleeper. Further, we observed a more remarkable peak around 320 Hz for the elastic vibration of the ballast layer compared with the actual measurement, which resulted from not only the coupled simulation of the ballast particle and sleeper, but also elastic deformation calculation of the ballast particles. Thus, we can confirm the associated behaviors of the elastic vibration of the ballast layer and the rigid/bending natural vibration of the sleeper by using QDEM simulation.

**Figure 12:**
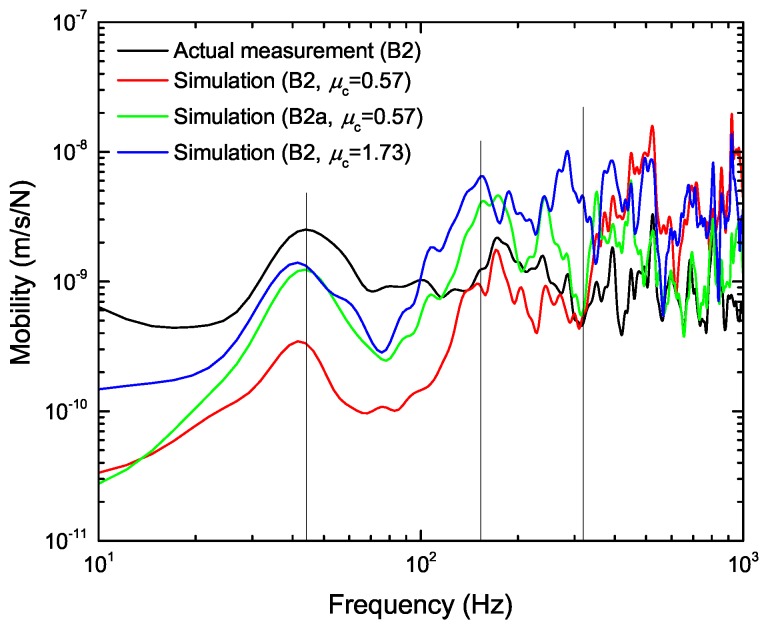
Frequency response function of ballast particle velocity under traffic impact load. The linear spectra are smoothed by applying a Parzen window with a bandwidth of 20 Hz.

Finally, we investigated the response of the ballast layer to the input traffic load. Figure 12 shows the mobility of the ballast particles at positions B2 and B2a, which is the amplitude of the frequency response function (FRF) obtained by the linear spectral function of the ballast particle velocity divided by that of the input load. The mobility indicates the risk of ballasted track deterioration in the frequency domain of the input load. Over a wide frequency domain, we confirmed that many peaks of the simulation fit the actual measurement. At a frequency of around 45 Hz, the ballast responds to the heavy wheel load applied to the rail during the passage of a train. On the other hand, each peak in the higher frequency region from 150 Hz represents the response for the high-frequency loading due to the rolling contact mechanism of the wheel on the rail. However, there is no peak in the resonance frequency domain of the ballast and sleeper around 320 Hz, which means that the vibration of the sleeper rather than the input load dominantly influences the ballast particle movement at this frequency. Moreover, compared with the result obtained under the low friction condition (red line), the ballast particle mobility with a high friction coefficient (blue line) becomes larger and approaches the actual measurement in the frequency region below 100 Hz. The ballast layer becomes stiff in the high friction condition because the ballast particles strongly connect with each other owing to the non-slip contact. Thus, we believe that the input load was effectively transmitted to each ballast particle through the ballast layer, which enabled the ballast to respond well to the input load, i.e., low frequency motion, because the ballast particles move together in a stiff connection, such as bonded particle material. Not only the frictional effect, but also the mobility differs considerably if the two ballast particles are neighbors, such as B2 and B2a, which depends on the ballast particle arrangement. Thus, we confirmed that friction and ballast particle arrangement influence the response of the ballast to the input load. The frictional contact motion of the ballast particle depends on not only the ballast particle arrangement, but also the contact calculation model considering its polyhedron shape; however, our contact model is currently too simple owing to computational speed considerations, as mentioned earlier. Therefore, improvement of the contact model might be necessary for further quantitative verification of the ballasted track motion, although the peak position of the response function was in good agreement with the actual measurement in a wide frequency range.

## 5. Advantages and Disadvantages of QDEM

From the verification of the fundamental behavior of linear viscoelastic materials and the investigation of deformable multi-body contact dynamics, we can summarize the remarkable features of QDEM simulation as follows.

- Advantages:
  − For elastic deformation analysis in both static and dynamic conditions, the computational accuracy is the same as that of FEM using a primary tetrahedron element.
  − For viscoelastic materials, the effect of the real shear viscosity on the attenuation of the vibration amplitude is effectively reproduced in dynamic analysis, and the effect of viscosity on creep-recovery motion can be qualitatively confirmed in quasi-static analysis.
  − The computational speed per time step and the parallel computing efficiency are much higher than those of FEM because massive matrix calculations are not necessary to obtain the viscoelastic stress of individual elements.
  − The rigid and elastic vibration characteristics can be analyzed via frictional contact dynamics of a large number of viscoelastic bodies, which cannot be accurately treated by DEM, such as the bonding particle method, and cannot be efficiently calculated by FEM.
- Disadvantages:
  − In comparison with implicit FEM, QDEM is a time-consuming method for simulating long-time events such as creep-recovery motion.
  − The accuracy of contact calculation between polyhedrons is currently not sufficient to consider the polyhedral shape effect because priority is given to the computational speed in order to deal with many bodies.
  − Fracture mechanics is not implemented, although the deterioration of the ballasted track might be influenced by the crushing or wearing of ballast particles owing to the impact load.

## 6. Conclusions

We developed QDEM, which is based on a particle method, for the deformation calculation of linear viscoelastic materials according to continuum mechanics by an explicit method. In QDEM, considering the viscoelastic stress on the particle volume element, the viscoelastic behavior can be simply obtained from only the information of four tetrahedron vertexes using the Kelvin–Voigt model.

To validate QDEM for the fundamental behavior of a linearly viscoelastic material, we simulated the static, quasi-static and dynamic behaviors of a real material with a geometry represented by connecting the viscoelastic tetrahedron. For bending analysis of an elastic cantilever, the displacement in a static state during loading and the vibration mode in a dynamic state after unloading were in good agreement with both the FE analysis and the analytical solution. The spatial distribution of the von Mises stress was also quantitatively equal to that of the FE analysis. On the other hand, for viscoelastic materials, the effect of viscosity on the creep and recovery behavior was confirmed, although the response was slightly slower than that of the analytical solution. Moreover, for the dynamic analysis of more rigid materials, QDEM reproduced the exponential decay of the vibration amplitude. The attenuation degree correlated well with the real physical viscosity. As a result, we verified that QDEM can reliably solve the typical dynamics of viscoelastic materials.

As an application of QDEM, ballasted track simulation was performed to investigate the multi-body contact dynamics of the ballast particles and sleeper. The ballast layer with higher friction was found to reduce the settlement of ballast particles against cyclic impact loads. In addition, the dominant vibration frequencies of the ballast particle and sleeper were observed around the natural frequency of the sleeper, as seen in actual measurements. In particular, we first reproduced the resonance mode of the sleeper vibration with the elastic vibration due to the stretching motion of the ballast layer by coupled simulation of the sleeper and ballast particles. Thus, we confirmed that QDEM can analyze the frictional contact dynamics of viscoelastic deformable multi-body systems, such as a ballasted track.

**Figure 13:**
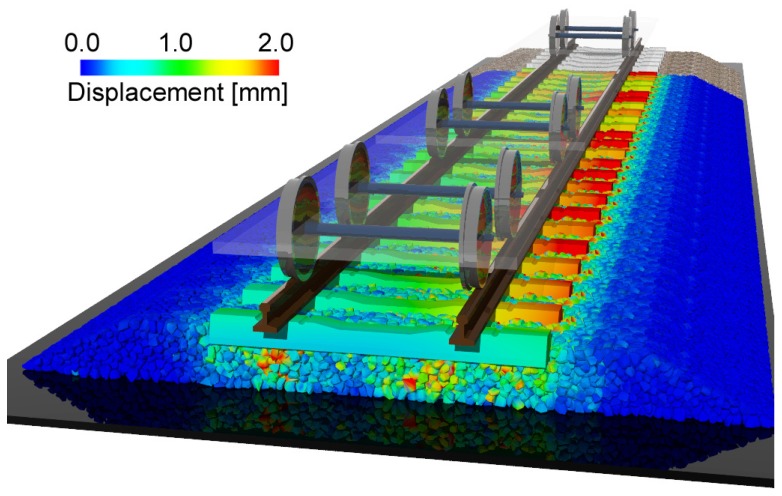
Snapshot of ballasted track dynamics simulation for a large-scale model of 24 sleepers using eight GPUs. The displacement is shown with a magnification of 20×. A video sequence of this simulation can be found as the Supplementary Material in Video S2.

In the future, a multi-GPU parallel computing system will be used to extend the QDEM features to large-scale simulation with many sleepers, as shown in Figure 13 (see also Video S2 in the Supplementary Materials). Moreover, the accuracy of contact force calculation for irregularly-shaped objects, such as ballast particles, will be improved by implementing the contact detection method between polyhedrons (not DEM-like spherical particles). Finally, we will investigate the stress propagation in the traveling direction of the train in greater detail and accordingly suggest the optimum railway construction technique to reduce the ballasted track deterioration due to traffic impact loads.

## Figures and Tables

**Table 1:** Simulation conditions of the ballast and sleeper.

Property	Ballast	Sleeper	Unit
Number of tetrahedral elements	622,591	284,241	-
Number of particles	199,469	55,565	-
Density	2700	2350	kg/m^3^
Young’s modulus	30	45	GPa
Poisson’s ratio	0.200	0.167	-
Spring constant for DEM (normal)	1.613	2.390	GN/m
Spring constant for DEM (tangential)	0.672	1.024	GN/m
Shear viscosity	10,000	10,000	Pa s
Bulk viscosity	0	0	Pa s
Friction coefficient	0.577	0.577	-
Discrete time step	0.1	0.1	μs
DEM particle diameter	0.024	0.024	m
Number of objects	5700	1	-

## Supplementary Materials

The following are available online at http://www.mdpi.com/1996-1944/10/6/615/s1: Figure S1: Mesh structure of concrete beam used in FEM and QDEM; Figure S2: Mesh structure of Type-3 pre-stressed concrete (PC) sleeper used in QDEM; Video S1: Ballast and sleeper motion under traffic impact loads; Video S2: Large-scale ballasted track simulation.
